# One-step enzyme-free dual electrochemical immunosensor for histidine-rich protein 2 determination†

**DOI:** 10.1039/d0ra08729g

**Published:** 2020-12-23

**Authors:** Ariamna María Dip Gandarilla, Matias Regiart, Mauro Bertotti, Juliane Correa Glória, Luís André Morais Mariuba, Walter Ricardo Brito

**Affiliations:** Department of Chemistry, Federal University of Amazonas Manaus Amazonas 69067-005 Brazil wrbrito@ufam.edu.br +55 92 981379920; Department of Fundamental Chemistry, Institute of Chemistry, University of São Paulo São Paulo 05508-000 Brazil matiasregiart@gmail.com +55 11 982885489; Leônidas and Maria Deane Institute, Oswaldo Cruz Foundation Manaus Amazonas 69057-070 Brazil

## Abstract

In the present work, we describe a novel one-step enzyme-free dual electrochemical immunosensor for the determination of histidine-rich protein 2 (Ag-*Pf*HRP2), a specific malaria biomarker. A gold electrode (GE) was functionalized with the *Pf*HRP2 antibody (Ab-*Pf*HRP2) using dihexadecyl phosphate (DHP) polymer as an immobilization platform. The Ab-*Pf*HRP2/DHP/GE sensor was characterized by cyclic voltammetry, electrochemical impedance spectroscopy, Fourier-transform infrared spectroscopy, scanning electron microscopy, and atomic force microscopy. The developed immunosensor was employed for indirect Ag-*Pf*HRP2 determination by differential pulse voltammetry (DPV) and electrochemical impedance spectroscopy (EIS). The linear range was 10–400 ng mL^−1^ and 10–500 ng mL^−1^ for EIS and DPV, while the limit of detection was 3.3 ng mL^−1^ and 2.8 ng mL^−1^, respectively. The electrochemical immunosensor was successfully applied for Ag-*Pf*HRP2 determination in human serum samples. Its performance was compared with an ELISA test, and good correspondence was achieved. The coefficients of intra- and inter-assay variations were less than 5%. The electrochemical immunosensor is a useful and straightforward tool for *in situ* malaria biomarker determination.

## Abstract 2

One-step enzyme-free dual electrochemical immunosensor for Malaria biomarker determination.

## Graphical abstract

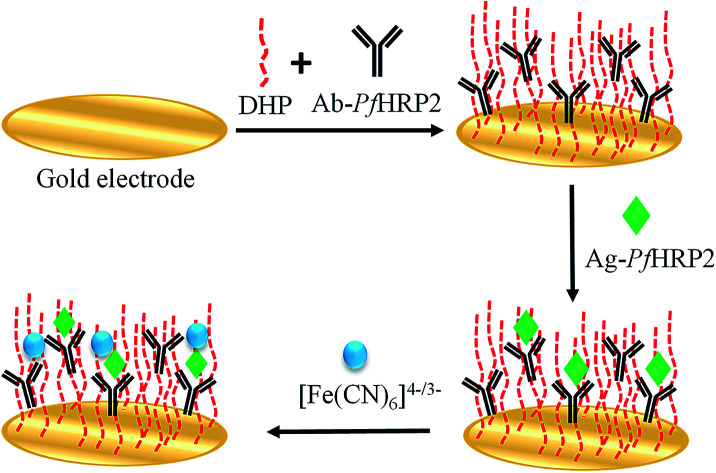

## 1. Introduction

Malaria is one of the most important tropical infectious parasitic diseases, caused by *Plasmodium* sp. parasites (*Plasmodium falciparum*, *ovale*, *vivax*, *malariae*, and *knowlesi*) and transmitted by the female of *Anopheles* mosquito. According to the World Malaria Report 2019, there was an estimate of 405 000 deaths globally in 2018, and the infections by *Plasmodium falciparum* parasite are the most common.^1^ The morbidity and mortality are higher in low resource populations, with limited health care facilities.^2^

Malaria diagnosis is commonly confirmed by microscopic examination, which consists of the observation of blood samples on a microscopic slide assisted with staining. However, such an approach requires trained professionals, specialized facilities, long analysis time, expensive equipment, and reagents.^3^ Nucleic acid amplification tests have also been used to detect the nucleic acid of the malaria parasite, including loop-mediated isothermal amplification, real-time quantitative polymerase chain reaction, and quantitative nucleic acid amplification techniques. However, they have many disadvantages, such as poor reproducibility at low concentrations, expensive and time-consuming, require good laboratory facilities, and skilled personnel.^4,5^ Rapid diagnostic tests (RDTs) based on the *Plasmodium* biomarkers detection are another alternative and hold advantages, like the low cost, short analysis times, the possibility of *in situ* multiple species detection, and require unskilled labor.^6^

Biomarkers are molecular, biochemical, or cellular variations measurable in biological samples that indicate any biological, pathogenic, or therapeutic response.^7^ Recent bibliography reported the development of several aptasensors and immunosensors for malaria based on biomarkers detection, like histidine-rich protein II (HRP-2),^8–10^ lactate dehydrogenase (LDH),^11–13^ aldolase (ALD),^14^ hypoxanthine-guanine phosphoribosyl transferase (HGPRT),^15^ glutamate dehydrogenase (GluDH),^16^ and products such as hemozoin.^17^

In the last years, nanotechnology advances allowed the integration of novel materials into bio-analytical detection systems based on recognition elements such as enzymes, antibodies, nucleic acids, among others.^18,19^ These devices are characterized by simple, rapid, and accurate results due to the selective and sensitive interaction between bio-reagents and target analytes without the need to remove interferants from the sample before detection. The application of such devices is extended to process monitoring, clinical diagnosis, and evaluation of environmental, food, and water safety.^20,21^

The immobilization step is an essential subject in immunosensors development to obtain a satisfactory performance and appropriate characteristics. Even though many protocols can be found in the literature where crosslinkers are used to perform a covalent link between the immunoreagents and the immobilization platforms, these immobilization processes require long time-consuming steps and entirely known reaction conditions.^9,22^ Another option for biomolecules immobilization is the entrapment into polymers networks.^23^ Dihexadecyl phosphate (DHP) is a polymeric surfactant widely used as an encapsulating material. This polymer has a phosphate group negatively charged in the polar head, connected to two long hydrocarbon chains, is hydrophobic, and does not form micelles. However, DHP can be dispersed in water and organic solvents using ultrasonic stirring, resulting in a homogeneous and stable dispersion with a gel-crystalline aspect.^24^ Such stable films have been used to develop electrochemical sensors and biosensors.^25–27^

In this study, we present a one-step enzyme-free electrochemical immunosensor to determine *Plasmodium falciparum* histidine-rich protein 2 antigen (Ag-*Pf*HRP2), using a first-time DHP as a polymer to immobilize *Pf*HRP2 antibody directly on a gold electrode (GE) surface. The Ab-*Pf*HRP2/DHP/GE was characterized by cyclic voltammetry (CV), electrochemical impedance spectroscopy (EIS), Fourier-transform infrared spectroscopy (FTIR), scanning electron microscopy (SEM), and atomic force microscopy (AFM). The immunosensor was employed for indirect Ag-*Pf*HRP2 determination by differential pulse voltammetry (DPV) and electrochemical impedance spectroscopy (EIS). The results were correlated with a conventional ELISA test, showing a good correspondence. This novel device is a low cost and simple tool for *in situ* malaria biomarker determination.

## 2. Materials and methods

### 2.1. Chemicals and reagents

DHP, potassium hexacyanoferrate(iii), and potassium chloride were purchased from Sigma-Aldrich (St. Louis, USA). Potassium hexacyanoferrate(ii) trihydrate, sulfuric acid (98%), and buffer saline solution (PBS) of pH 7.00 were from Merck (Darmstadt, Germany). Purified polyclonal Ab-*Pf*HRP2, Ag-*Pf*HRP2, and human serum samples were obtained from Leônidas and Maria Deane Institute (Oswaldo Cruz Foundation, Manaus, Brazil). The biomolecules preparation process consisted of the recombinant expression of *Plasmodium falciparum* HRP2 in *Escherichia coli* BL21 pLysS (Invitrogen), and the purification was performed using affinity chromatography nickel columns (QIAGEN), following the manufacturer instructions.^28^ Later, mice were immunized with the purified recombinant protein (4 bi-weekly 10 μg doses). Serum samples were collected, and reactivity was analyzed by indirect enzyme-linked immunosorbent assay (ELISA). Antibodies were purified using the protein G sepharose (Sigma) resin column following the manufacturer's recommendations.

All the other reagents employed were of analytical grade and were used without further purification. Aqueous solutions were prepared by using purified water from a Milli-Q system.

### 2.2. Equipment

All electrochemical measurements (CV, EIS, and DPV) were performed using a PGSTAT128N potentiostat from Methrohm Autolab, with a NOVA 1.11 electrochemical analysis software. A conventional three-electrode system was used with a gold electrode (GE) (2 mm in diameter) as the working electrode, Ag/AgCl as the reference electrode (sat. KCl), and platinum as the auxiliary electrode.

All pH measurements were made with a precision pH meter (F20, FiveEasy). An ultrasonic bath (Q3350, QUIMIS) was also used.

The chemical groups were identified by Fourier-transform infrared spectroscopy (FTIR), coupled with an attenuated total reflectance (ATR) accessory (Agilent, Cary 630 Model). The number of scans was set to 150 and the resolution to 8.

Scanning electron microscopy images were taken on a VEGA3, TESCAN. Atomic force microscopy measurements were carried out on a Nanosurf Lens AFM instrument, equipped with a c3000 controller, using tapping contact mode and commercial tips (TAP 190, Al-G).

### 2.3. Immunosensor preparation

To improve the measurement's reproducibility, the bare GE (2 mm in diameter) was polished with 0.3 and 0.05 μm alumina slurry, polishing sandpaper (P4000), and cloth pad from Buehler (Illinois, USA), followed by ultrasonication in deionized water for 3 minutes. An electrochemical cleaning step was then performed by cyclic voltammetry between +0.0 and +1.5 V (*vs.* Ag/AgCl, sat. KCl) in 0.5 mol L^−1^ H_2_SO_4_ solution, with a scan rate of 50 mV s^−1^. Afterward, the electrode was rinsed with water and dried using an argon gas stream. A DHP solution (1 μg μL^−1^) was then prepared in 0.1 mol L^−1^ PBS pH 7.00 under ultrasonic stirring for two hours.^29^ The antibody was coated by adding 3 μL of an Ab-*Pf*HRP2 (0.05 μg μL^−1^)/DHP (1 μg μL^−1^) solution onto the electrode surface. The fabricated immunosensor was allowed to dry for 12 hours at 4 °C.

### 2.4. Determination of Ag-*Pf*HRP2

**Scheme 1:**
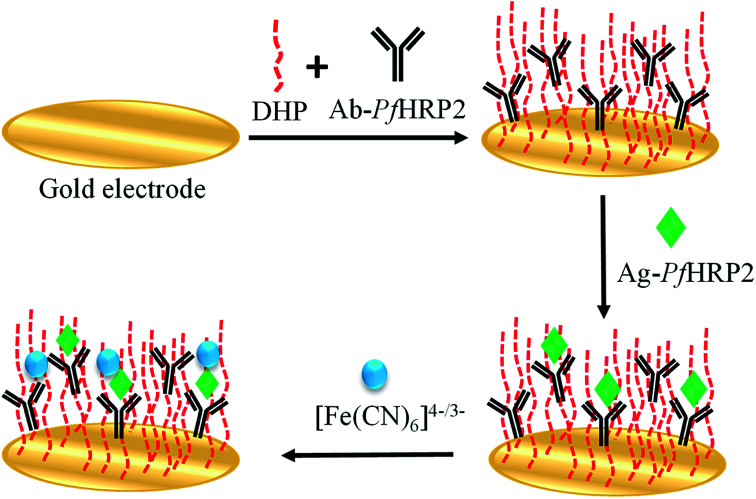
Schematic immunosensor fabrication and analytical procedure for Ag-*Pf*HRP2 determination.

Firstly, the immunosensor was incubated in the sample solution (previously diluted 1/50 in 0.1 mol L^−1^ PBS pH 7.00) for 40 minutes. In this step, the antigen present in the sample binds to the antibody. Later, a washing step with 0.1 mol L^−1^ PBS pH 7.00 was performed. Finally, the dual detection step was carried out in a 5 mmol L^−1^ [Fe(CN)_6_]^4−^/^3−^ + 0.1 mol L^−1^ KCl solution by differential pulse voltammetry measurements from −0.1 to +0.7 V at a scan rate of 25 mV s^−1^ under modulation amplitude of 50 mV, and by electrochemical impedance spectroscopy, at +0.23 V, varying the frequency with logarithmic spacing frequency in the range from 10 mHz to 100 kHz (Scheme 1).

### 2.5. Ethical review

This study was approved by the Brazilian Animal Ethical Committee (CEUA-UFAM 005/2010) and by the Human Research Ethical Committee of Amazonas Federal University (CAAE 00622218.3.0000.5020).

## 3. Results and discussion

### 3.1. Chemical and morphological characterization

**Fig. 1:**
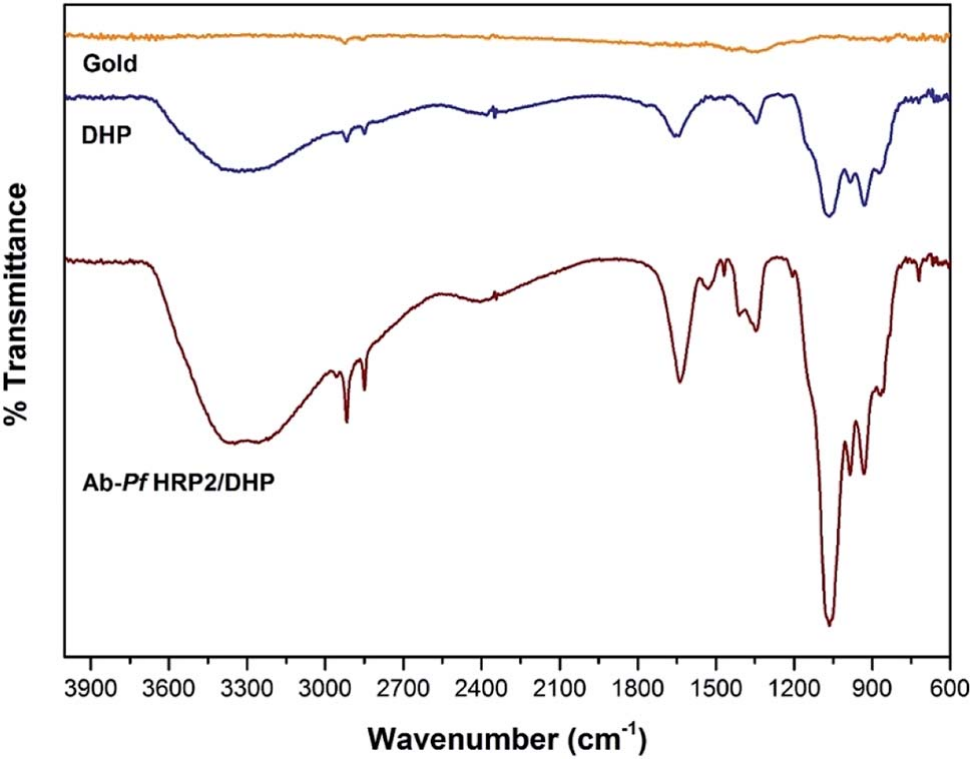
ATR-FTIR of the surfaces of bare GE, GE modified with DHP, and GE modified with Ab-*Pf*HRP2/DHP.

The chemical environment of the modified gold electrode surface was characterized by the FTIR technique. Fig. 1 shows the C–H stretching vibrations at 2916 and 2847 cm^−1^, and C–H bend vibrations at 1342 cm^−1^, corresponding to the DHP aliphatic chains. Peaks around 2384 and 1648 cm^−1^ were observed and attributed to a phosphate group (P–O–H stretching and bending vibration, respectively). The peak at 1066 cm^−1^ corresponds to the P–O–C stretch vibration.^30^ The antibody presence is associated with vibrations resulting from O–H and N–H stretching bands (3200–3400 cm^−1^) and the increase in the signal intensity at 1648 and 1066 cm^−1^ due to C=O and C–O bonds. These signals are characteristics of typical amino acids present in proteins.

**Fig. 2:**
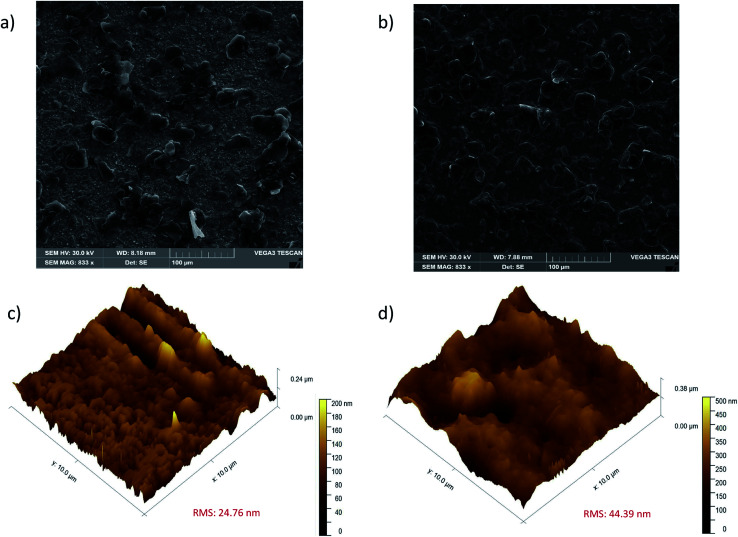
SEM and 3-dimensional AFM images: (a and c) DHP/GE surface, (b and d) Ab-*Pf*HRP2/DHP/GE surface.

The morphological characterization of the modified GE with DHP and Ab-*Pf*HRP2/DHP was conducted employing SEM and AFM techniques. Fig. 2a shows the granular structures of DHP on the GE surface, while the electrode surface after Ab-*Pf*HRP2/DHP deposition reveals surface densification (Fig. 2b).

AFM images of the topography of DHP and Ab-*Pf*HRP2/DHP modified electrode surfaces are presented in Fig. 2c and d, respectively. The 24.76 nm and 44.39 nm root mean square roughness (RMS) values for the DHP and Ab-*Pf*HRP2/DHP films, respectively, indicated the successful modification of the electrode surface. By inspection of such figures, one can also conclude that the Ab-*Pf*HRP2/DHP film exhibits a more irregular and rougher surface compared to that of DHP.

### 3.2. Electrochemical characterization

**Fig. 3:**
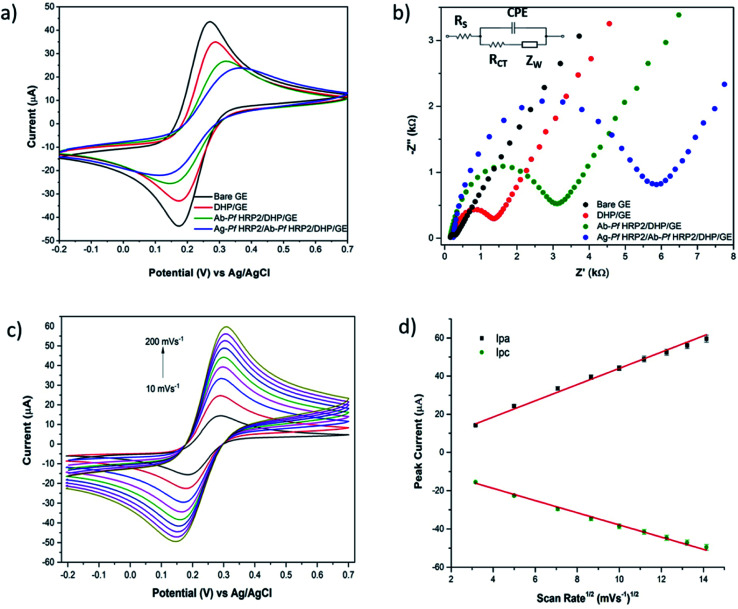
(a) CVs recorded between −0.2 and +0.7 V at 50 mV s^−1^, (b) EIS recorded at +0.23 V by varying the frequency with logarithmic spacing frequency in the range from 10 mHz to 100 kHz, (c) CVs recorded with the immunosensor at different scan rates (10, 25, 50, 75, 100, 125, 150, 175, 200 mV s^−1^), and (d) plots of peak currents as a function of the square root of the scan rate. All the electrochemical measurements were carried out in 5 mmol L^−1^ [Fe(CN)_6_]^4−/3−^ + 0.1 mol L^−1^ KCl solution.

The GE surface modification was examined by CV and EIS. The CV of a soluble electroactive species with fast, reversible electrochemical behavior is an invaluable tool for monitoring several stages in the electrode surface modification. CVs were recorded in 5 mmol L^−1^ [Fe(CN)_6_]^4−^/^3−^ + 0.1 mol L^−1^ KCl solution, in a potential range between −0.2 and +0.7 V at 50 mV s^−1^. As can be seen in Fig. 3a, the bare GE shows a well-defined reversible response for [Fe(CN)_6_]^4−^/^3−^, confirming the electron transfer is mass-transport controlled at the bare electrode. On the other hand, the ability of the redox probe to access the electrode surface decreases for the modified electrode with DHP because such a compound is negatively charged and hinders the diffusion of ferricyanide/ferrocyanide towards the electrode surface by charge exclusion.^31^ The voltammetric profile obtained with the Ab-*Pf*HRP2/DHP and Ag-*Pf*HRP2/Ab–*Pf*HRP2/DHP modified electrodes followed a similar trend, *i.e.*, broader voltammograms with larger anodic–cathodic peak separation, indicating partially blocked electron transfer because of the insulating characteristics of the immobilized biological structures.

EIS experiments were carried out in 5 mmol L^−1^ [Fe(CN)_6_]^4−^/^3−^ + 0.1 mol L^−1^ KCl, within the conditions previously reported. The obtained EIS data were represented as Nyquist (Fig. 3b) and Bode (Fig. S1†) plots. The impedance spectrum includes a semicircle at higher frequencies representing the electron transfer resistance, evidencing the blocking behavior of the bare/modified electrode surface towards the redox probe, and a linear part at lower frequencies that represents the diffusion process. Resistance values (*R*_ct_) were evaluated by iterative fitting (NOVA 1.11 software) of the experimental data to the modified Randles equivalent circuit (Fig. 3b inset), where *R*_s_ is the solution resistance, *Z*_w_ is the Warburg impedance, and CPE is the constant phase element. As it can be observed, lower electron transfer resistance values were obtained with the bare gold electrode (136 Ω). The semicircle increased (*R*_ct_ = 1.21 kΩ) due to the increase of the insulating layer thickness resulting from the DHP polymer deposition process. This behavior has been previously reported for gold,^31^ and glassy carbon^32^ electrodes modified with DHP and was attributed to the partial blockage of the electron transfer. The immobilization of large molecular antibody structures increased the gold surface's insulator behavior and led to an increase in the semicircle curve (*R*_ct_ = 2.82 kΩ), confirming the successful Ab-*Pf*HRP2 deposition.^33^ Finally, an even higher hindrance effect of the antibody–antigen complex was noticed after attachment of the antigens (100 ng mL^−1^ Ag-*Pf*HRP2) to the immobilized antibodies, resulting in a significant R_ct_ increase (up to 5.29 kΩ).

The electron transfer behavior of [Fe(CN)_6_]^4−^/^3−^ redox couple at the Ab-*Pf*HRP2/DHP/GE immunosensor was investigated by performing cyclic voltammetry experiments varying the scan rates from 10 to 200 mV s^−1^ in the potential range between −0.2 and +0.7 V. Fig. 3c and d show that both anodic (*I*_pa_) and cathodic (*I*_pc_) peak currents increased linearly with the square root of the scan rate (*v*^1/2^), suggesting that the electrochemical reaction was a diffusion-controlled process.^34^ The regression equations of the two straight lines are as follows: *I*_pa_ (μA) = 4.255*v*^1/2^ (mV s^−1^) + 1.496 (*R*^2^ = 0.992), *I*_pc_ (μA) = −3.183*v*^1/2^ (mV s^−1^) − 6.053 (*R*^2^ = 0.995).

An immunosensor stability test was conducted by recording cyclic voltammograms after storage for one week in a moist chamber at 4 °C (Fig. S2†). The results showed a similar profile in both voltammograms (before and after storage), with a slight modification in peak potentials without significant variation in the peak currents (*I*_pa_ and *I*_pc_), indicating good stability.

### 3.3. Optimization of analytical parameters

**Fig. 4:**
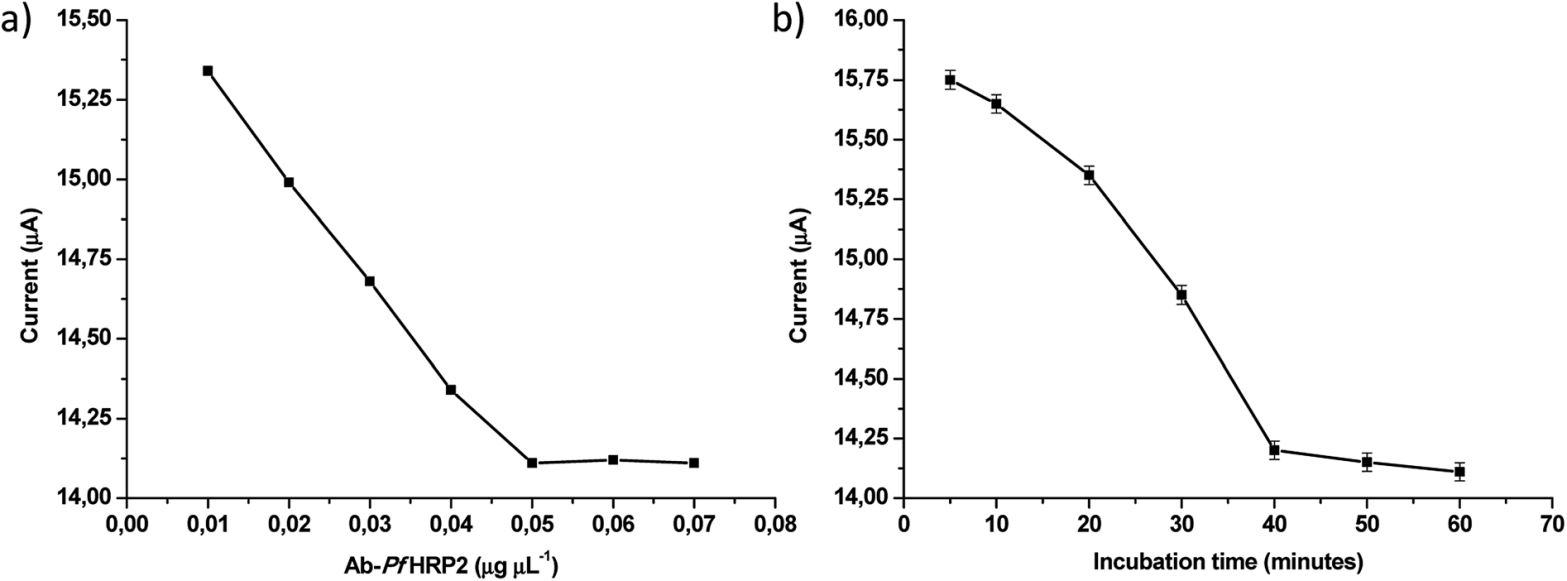
(a) Optimization of Ab-*Pf*HRP2 immobilization concentration, (b) optimization of Ag-Ab-*Pf*HRP2 incubation time.

The influence of experimental parameters that affect the Ab-*Pf*HRP2/DHP/GE electrochemical immunosensor performance in biological samples was investigated in a 100 ng mL^−1^ Ag-*Pf*HRP2 standard solution using DPV. The Ab-*Pf*HRP2 concentration in the solution employed for the immobilization into the DHP modified electrode surface was optimized. The concentration was varied from 0.01 to 0.07 μg μL^−1^ Ab-*Pf*HRP2, and the current response decreased with the increase in the antibody concentration until 0.05 μg μL^−1^ Ab-*Pf*HRP2 (Fig. 4a). No further current decrease was noticed at higher Ab-*Pf*HRP2 concentrations, hence 0.05 μg μL^−1^ Ab-*Pf*HRP2 was adopted as optimum concentration for the Ab-*Pf*HRP2 immobilization.

The Ag-Ab-*Pf*HRP2 incubation time was also optimized in the interval from 10 to 60 minutes, and the results are shown in Fig. 4b. A continuous decrease in the current response was noticed up to 40 minutes of incubation, and the signal remained constant for longer times. Accordingly, 40 minutes was used for antigen–antibody binding as the optimum interaction time between the immune reagents.

Other parameters such as optimum pH for the analytical determination and DHP polymer concentration employed for the Ab-*Pf*HRP2 immobilization were used, according to Ardila *et al.*^29^

### 3.4. Immunosensor analytical performance

**Fig. 5:**
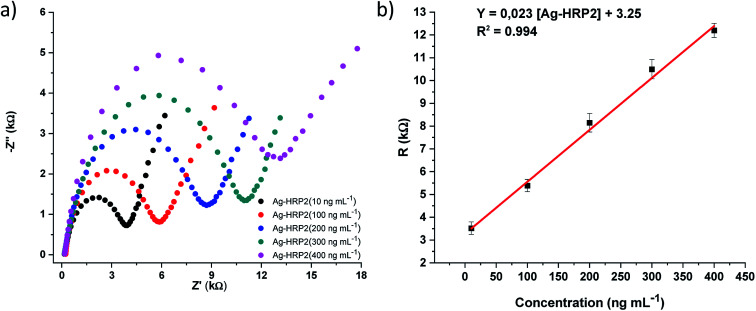
(a) EIS spectra recorded with the Ab-*Pf*HRP2/DHP/GE electrochemical immunosensor upon additions of Ag-*Pf*HRP2 (10, 100, 200, 300, and 400 ng mL^−1^), (b) calibration curve: *Y* = 0.023 [Ag-*Pf*HRP2] + 3.25, with an *R*^2^ = 0.994.

The analytical performance of the immunosensor was examined with Ag-*Pf*HRP2 standard solutions (*n* = 8) at different concentrations: 1, 10, 100, 200, 300, 400, 500, and 750 ng mL^−1^ in 0.1 mol L^−1^ PBS pH 7.00. Fig. 5 shows the EIS spectra for several Ag-*Pf*HRP2 concentrations from 10 to 400 ng mL^−1^. The calibration curve was obtained by plotting resistance (*R*) (kΩ) *versus* Ag-*Pf*HRP2 concentration (ng mL^−1^). The calibration curve was defined as *R* (kΩ) = 0.023 [Ag-*Pf*HRP2] + 3.25, with *R*^2^ = 0.994. The coefficient of variation (CV) for the determination of 100 ng mL^−1^ Ag-*Pf*HRP2 was 4.95% (*n* = 5).

**Fig. 6:**
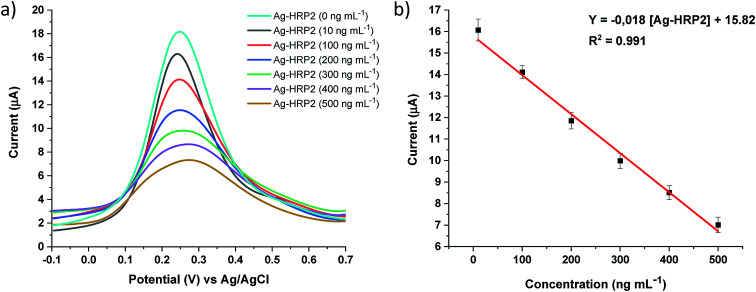
a) DPV recorded with the Ab-*Pf*HRP2/DHP/GE electrochemical immunosensor upon additions of Ag-*Pf*HRP2 (10, 100, 200, 300, 400, and 500 ng mL^−1^), (b) calibration curve: *Y* = −0.018 [Ag-*Pf*HRP2] + 15.82, with an *R*^2^ = 0.991.

Similar experiments were repeated using DPV as a detection technique, and a linear relationship was observed from 10 to 500 ng mL^−1^ Ag-*Pf*HRP2. The calibration curve was obtained by plotting current (μA) *versus* Ag-*Pf*HRP2 concentration (ng mL^−1^) and was defined by current (μA) = −0.018 [Ag-*Pf*HRP2] + 15.82, with *R*^2^ = 0.991 (Fig. 6). The coefficient of variation (CV) for the determination of 100 ng mL^−1^ Ag-*Pf*HRP2 was 4.88% (*n* = 5).

Moreover, results obtained with the electrochemical immunosensor were compared with those of an ELISA test (ESI 3†). The ELISA test showed a linear relationship in the concentration range from 15 to 125 ng mL^−1^ Ag-*Pf*HRP2. The calibration curve was obtained by plotting optical density (OD) *versus* Ag-*Pf*HRP2 concentration (ng mL^−1^) and was defined by OD = 0.01 [Ag-*Pf*HRP2] + 0.24, with *R*^2^ = 0.993 (Fig. S3a†). The coefficient of variation (CV) for the determination of 100 ng mL^−1^ Ag-*Pf*HRP2 was 5.44% (*n* = 5).

To evaluate the correlation between both methods, Ag-*Pf*HRP2 responses for concentrations from 20 to 120 ng mL^−1^ were compared, and a linear behavior with a slope of 1.008 was found, indicating good correspondence between the ELISA and the electrochemical immunosensor (EIS detection) (Fig. S3b†).

The detection limit (LOD) was calculated according to the IUPAC recommendations.^35^ The LOD was found to be 3.3 ng mL^−1^ and 2.8 ng mL^−1^ for EIS and DPV, respectively, while the LOD for the ELISA test was 5.5 ng mL^−1^. The total assay time for the Ag-*Pf*HRP2 determination was 45 minutes, much less than the one usually required for the conventional ELISA test (180 minutes).^36^

To evaluate the analytical applicability of the electrochemical immunosensor, Ag-*Pf*HRP2 was quantified in seven negative spiked human serum samples by DPV and EIS techniques. Results were compared with those obtained with the ELISA test. A good correlation between results for spiked Ag-*Pf*HRP2 samples can be observed in Table 1. Hence, the developed immunosensor can be considered as selective towards the interferences present in the samples. The dynamic concentration range for Ag-*Pf*HRP2 detection using the electrochemical immunosensor is very large, therefore the device is useful to monitor both non-severe and severe malaria, as values greater than 100 ng mL^−1^ are typically associated with severe malaria.^37,38^

**Table 1:** Comparison of Ag-*Pf*HRP2 concentration in human serum samples by EIS and DPV using the electrochemical immunosensor and the ELISA test (average of three determinations ± SD)

Samples^a^	EIS^b^	DPV^c^	ELISA
0	0.01 ± 0.01	0.01 ± 0.01	0.02 ± 0.02
10	10.1 ± 0.5	10.2 ± 0.6	9.5 ± 0.4
50	50.3 ± 0.1	49.9 ± 0.2	51.9 ± 0.3
100	99.8 ± 0.5	99.8 ± 0.6	102.5 ± 0.6
200	201.1 ± 1.1	199.8 ± 1.2	197.6 ± 1.5
300	302.2 ± 1.3	301.3 ± 1.4	303.2 ± 1.8
400	399.1 ± 2.1	402.8 ± 2.1	403.5 ± 2.5

a Ag-*Pf*HRP2 human serum samples (ng mL^−1^).

b Electrochemical impedance spectroscopy.

c Differential pulse voltammetry.

## 4. Conclusions

The developed electrochemical immunosensor for Ag-*Pf*HRP2 detection offered several attractive advantages like good stability, high selectivity, and sensitivity. The applied analytical method is based on a novel one-step enzyme-free dual electrochemical immunosensor performed by the simple Ab-*Pf*HRP2 immobilization on a gold electrode surface using dihexadecyl phosphate polymer as an immobilization platform. Compared with the conventional method used to detect Ag-*Pf*HRP2, an ELISA test, the electrochemical immunosensor shows better analytical properties, like a wide linear range and a low detection limit. The immunosensor was successfully applied for the Ag-*Pf*HRP2 determination in human serum samples, and the coefficients of intra- and inter-assay variations were less than 5%. The total assay time employed was four times shorter than the time reported for the frequently used ELISA test. The electrochemical immunosensor can be a useful and straightforward tool for biomedical sensing and clinical applications for *in situ* diagnosis and prognosis of a malaria biomarker determination in human serum samples.

## Authorship contributions

Ariamna María Dip Gandarilla: conceptualization, investigation, validation, data curation, writing-original draft. Matias Regiart: methodology, investigation, data curation, writing review & editing. Mauro Bertotti: supervision, writing review & editing. Juliane Correa Glória: methodology, data curation, validation. Luís André Morais Mariuba: investigation, supervision. Walter Ricardo Brito: conceptualization, supervision, writing review & editing, project administration.

## Conflicts of interest

There are no conflicts to declare.

## Supplementary Material

RA-011-D0RA08729G-s001
