# Supplementary material for: One-step enzyme-free dual electrochemical immunosensor for histidine-rich protein 2 determination
Source: RSC Adv. 2020 Dec 23;11(1):408–15. doi: 10.1039/d0ra08729g (PMC8691096; doi:10.1039/d0ra08729g)
Supplement: RA-011-D0RA08729G-s001 [file RA-011-D0RA08729G-s001.pdf]

Electronic Supplementary Material (ESI) for RSC Advances.
This journal is © The Royal Society of Chemistry 2020

One-step enzyme-free dual electrochemical immunosensor for Histidine-Rich Protein 2 determination

Supplementary Information

Ariamna María Dip Gandarilla[a], Matias Regiart[b*], Mauro Bertotti[b], Juliane Correa Glória[c], Luís André Morais Mariuba[c], Walter Ricardo Brito[a*].

[a] *Department of Chemistry, Federal University of Amazonas, Manaus, Amazonas 69067-005, Brazil.*

[b] *Department of Fundamental Chemistry, Institute of Chemistry, University of São Paulo, São Paulo 05508-000, Brazil.*

[c] *Leônidas and Maria Deane Institute, Oswaldo Cruz Foundation, Manaus, Amazonas 69057-070, Brazil.*

*Corresponding authors: Walter Ricardo Brito +55 92 981379920 *(wrbrito@ufam.edu.br)*, Matias Regiart +55 11 982885489 *(matiasregiart@gmail.com)*

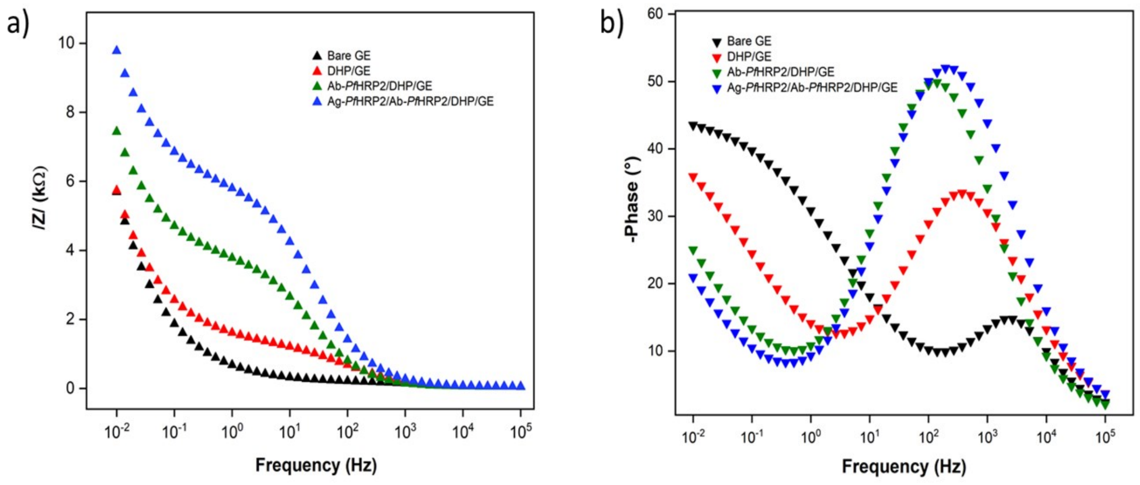

Fig. SI 1. Bode plot impedance a) and phase b) of the electrode surface modifications.

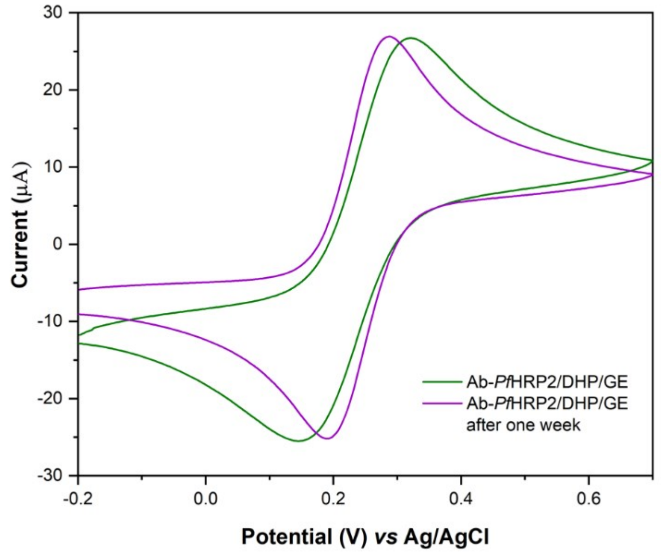

Fig. SI 2. Cyclic voltammograms of immunosensor before and after storage during one week at 4 °C.

SI 3. ELISA test

ELISA 96-well microtiter plates (NUNC/Marxisorp) were sensitized for 16 hours at 4 °C in a humid chamber, with 50 μL per well of a solution containing 4 μg mL^{-1} *Pf*HRP2 mice antibodies diluted in 0.05 mol L^{-1} carbonate-bicarbonate buffer pH 9.6. After this period, the plates were washed with PBS containing 0.05 % Tween 20 and then blocked for 1 hour at 37 °C with PBS containing 5 % bovine serum albumin. Then, recombinant

*Pf*HRP2 protein was added in different concentrations and the resulting solutions were incubated for 1 hour at 37 °C. Afterward, the *Pf*HRP2 chicken antibodies were added at a concentration of 0.25 µg per well. These plates were incubated for 1 hour at 37 °C in a humid chamber, followed by three washes with PBS containing 0.01 % Tween 20. Next, goat anti-chicken IgG antibody conjugated with horseradish peroxidase (KPL) was added at 1/5000 dilution and incubated again for 1 hour at 37 °C in a humid chamber. After a new cycle of three washes, 50 µL of one-step reagent (SCIENCO) were added to the plate and incubated for 20 minutes at room temperature in the absence of light. The reaction was stopped with 50 µL of 1 mol L^{-1} H_2SO_4. The generated product was quantified by reading the optical density (O.D.) at 450 nm in a spectrophotometer (FlexStation3, Molecular Devices).

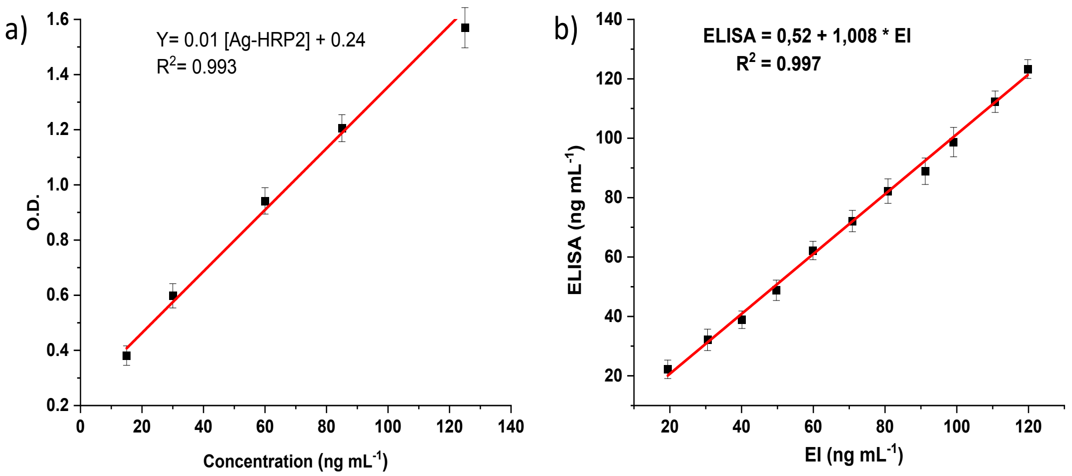

Fig. SI 3. a) ELISA calibration curve: Y = 0.01 [Ag-*Pf*HRP2] + 0.24, with an R^2 = 0.993. b) Correlation between the ELISA and the electrochemical immunosensor by EIS detection.
